# Population structure, genetic diversity and pathotypes of *Streptococcus suis* isolated during the last 13 years from diseased pigs in Switzerland

**DOI:** 10.1186/s13567-020-00813-w

**Published:** 2020-07-08

**Authors:** Simone Scherrer, Giuliana Rosato, Nathalie Spoerry Serrano, Marc J. A. Stevens, Fenja Rademacher, Jacques Schrenzel, Marcelo Gottschalk, Roger Stephan, Sophie Peterhans

**Affiliations:** 1 Department of Veterinary Bacteriology, Institute for Food Safety and Hygiene, Vetsuisse Faculty, University of Zurich, Zurich, Switzerland; 2 Institute for Veterinary Pathology, Vetsuisse Faculty, University of Zurich, Zurich, Switzerland; 3 Bacteriology Laboratory, Geneva University Hospitals and University of Geneva, Geneva, Switzerland; 4 Swine and Poultry Infectious Diseases Research Center (CRIPA), Groupe de recherche sur les maladies infectieuses des animaux de production (GREMIP), Faculty of Veterinary Medicine, University of Montreal, Saint-Hyacinthe, QC Canada

**Keywords:** *Streptococcus suis*, MLST, Capsular type, Clonal complex, Virulence potential, Invasive disease-associated, MCG groups

## Abstract

*Streptococcus* (*S.*) *suis* is a globally important swine pathogen, which comprises certain zoonotic serotypes. In this study, a detailed characterization of 88 porcine *S. suis* isolates was performed by analyzing capsular (*cps*) types, multilocus sequence typing (MLST) and investigation of the minimum core genome (MCG). In order to focus on the virulence potential of presumable invasive disease-associated *S. suis* isolates, virulence-associated gene profiles were assessed followed by screening a chosen subset of *S. suis* strains with a molecular pathotyping tool. Results showed a high genetic variability within this strain collection. In total, seventeen *cps* types were identified with a predominance of *cps* type 9 (15.9%) and 6 (14.8%). MLST revealed 48 sequence types (STs) including 41 novel ones. The population structure of *S. suis* was heterogenous and isolates belonged to eight different clonal complexes (CCs) including CC28 (9.1%), CC1109 (8%), CC13/149 (6.8%), CC1237 (5.7%), CC1 (3.4%), CC17 (3.4%), CC87 (2.3%), and CC1112 (1.1%), whereas a significant portion of isolates (60.2%) could not be assigned to any described CCs. Virulence-associated markers, namely extracellular protein factor (*epf*), muramidase-released protein (*mrp*), and suilysin (*sly*), showed a link with STs rather than with *cps* types. With this study an expanded knowledge about the population structure and the genetic diversity of *S. suis* could be achieved, which helps to contribute to an optimal public health surveillance system by promoting a focus on strains with an increased virulence and zoonotic potential.

## Introduction

*Streptococcus suis* (*S. suis*) is a Gram-positive bacterium recognized to be an important swine pathogen responsible for various diseases including septicemia with sudden death, meningitis, endocarditits, polyserositis, and arthritis [1]. *S. suis* also colonizes the upper respiratory tract with commensal strains giving rise to carrier pigs [2]. Moreover, *S. suis* can appear as an opportunistic pathogen in combination with other pathogens [3]. In addition, infection with *S. suis* not only leads to major economic problems due to important losses in pig production worldwide, but the bacterium is also capable of infecting humans. In Western countries, infection of humans usually involves a single person such as pig farmers, veterinarians, people who work at abattoir, or butchers. Infection mainly takes place through direct contact of skin wounds with contaminated pork even in cases where no obvious wound is present [4]. On the other hand, in Southeast Asian countries, the main route of infection seems to be the gastrointestinal tract where consumption of undercooked pork products or a traditional soup containing raw pig blood is common and humans frequently live in close contact to pigs [5]. The most frequently described manifestations in humans are meningitis and septic shock [6].

Currently, there are at least 29 *S. suis* serotypes [7] described based on a serological reaction against the capsular polysaccharide, which has been commonly reported to be a major virulence factor with antiphagocytic properties [8]. Strains of heterogeneous serotype 2, which are responsible for more than 80% of human cases, represent a globally emerging zoonotic threat [9]. To a lesser extent, serotype 14 is also associated with disease in both humans and pigs [10]. Worldwide, major *S. suis* serotypes isolated from diseased pigs are serotypes 2, 9, 3, 1/2 and 7 [9]. In some European countries, serotype 9 strains are emerging and are considered to be responsible for causing disease among pigs [11] and sporadically capable to infect humans as reported in one case report from Thailand [12].

The most commonly described virulence markers for *S. suis* include muramidase-released protein (MRP) [13], extracellular protein factor (EF) [14], and the hemolysin suilysin (SLY) [15], which were mainly associated with a virulence potential of *S. suis* serotype 2 strains [16]. MRP is a surface protein anchored to the cell wall [17] and has recently been shown to be a major fibrinogen-binding protein [18]. Human fibrinogen bound to MRP increases viability of *S. suis* serotype 2 in human blood and its capability of migrating across the human cerebral endothelial cell barrier, thereby promoting the development of meningitis [19]. EF is secreted independent of an interaction between bacteria and host cell involved in enhancing bacterial infection, invasion, and pathogenicity [20]. Both MRP and EF have variants of different molecular weights [21, 22]. Strains harboring the short form of *epf* are considered the most virulent form of the *epf* subtypes [21]. SLY is a thiol-activated toxin antigenically related to cholesterol-binding toxins, which forms transmembrane pores [23] and is described to be toxic to phagocytic, endothelial, and epithelial cells [24]. However, since isogenic mutants can be found, which do not express MRP, EFP, and/or SLY and are still virulent, it is more precise to term those factors as virulence-associated markers [14].

Multilocus sequence typing (MLST) is a useful method to examine the population structure and provide epidemiological tracing of pathogenic bacteria. It has been applied to various species and is a valuable tool that allows further investigation of the global distribution and evolution of several pathogens [25], including *S. suis* as reported in former studies [26, 27]. The classification of sequence types (STs) resulting from MLST allows grouping of genetically similar genotypes in clonal complexes (CCs), thus revealing the population structure of *S. suis*. Various CCs are identified globally, whereas the prevalence of certain genotypes can vary in different geographical regions. The most important CCs implicated in human infections are the following: CC1 (harboring most important ST1) in Europe, Asia, North- and South America, and Africa [28]; CC20 in the Netherlands [11]; CC25 and CC28 in North America, Australia, and Asia [28], although in the United States an increasing trend of the genetically diverse CC94 was observed [29]. In Europe, a high prevalence of virulent *mrp *+ *epf *+ *sly *+ *cps2* strains being part of CC1 and mrp*cps9 strains of CC16/87 can be noticed [30].

A molecular surveillance tool to predict the virulence potential of *S. suis*, by differentiating potentially invasive disease-associated strains from commensal or possibly opportunistic non-disease-associated strains is of advantage. Such a pathotyping tool was recently described by Wileman et al. [31] and, provided that its application is successful, could be used as a convenient and promising diagnostic assay enabling the detection of highly virulent emerging strains. Based on whole genome sequencing data, three pathotyping markers were identified [31]. First of all, a disease-associated marker was described, which is predicted to be a copper-exporting ATPase (SSU207) potentially playing an essential role in copper homeostasis in Gram-positive and -negative bacterial pathogens [31, 32]. The second proposed disease-associated marker is annotated as type I restriction-modification (RM) system S protein (SSU1589) in *S. suis* strain P1/7 (GenBank accession number NC_012925.1) and is supposed to be implicated in the defense of host bacteria [33]. The third genetic marker is thought to be non-disease-associated and the corresponding protein is suggested to be a putative sugar ATP-binding cassette (ABC) transporter (SSUST30534).

The aim of this study was to determine the genetic diversity of Swiss porcine *S. suis* isolates collected during the past 13 years and to assess their virulence potential. Observed phenotypes arising from pathological, histological and bacteriological examinations were compared to the virulence-associated genotype profiles obtained by analyzing the classical virulence-associated markers (*mrp, efp* and *sly*) and by classifying the *S. suis* population using a system based on minimum core genome (MCG). Moreover, a selected subset of *S. suis* strains, based on a comprehensive data availability of histopathological findings of the corresponding clinical cases, was screened with an available novel pathotyping tool [31]. The identification of major pathogenic strains could help to establish an optimal public health surveillance system by promoting a focus on strains with an increased virulence and zoonotic potential.

## Materials and methods

### *Streptococcus suis* isolates

In this study a total of 88 porcine isolates obtained from routine diagnostic submissions to the Department of Veterinary Bacteriology at the Vetsuisse Faculty, University of Zurich, within the time span of 2006–2019, were included. The samples were recovered from 77 independent farms located in three main regions of Switzerland (Central- and Eastern part, and Canton of Zurich) with one to three samples per farm always derived from different animals. No isolates came from the French speaking Western part of Switzerland and the Canton of Bern. Isolates of the same farm were included only if STs were different. Samples analyzed originated from the following sites: 34.1% (*n* = 30/88) from blood, 20.5% (*n* = 18/88) from brain, 14.8% (*n* = 13/88) from lung, 11.4% (*n* = 10/88) from joint, 8% (*n* = 7/88) from heart, 3.4% (*n* = 3/88) from genital tract, 2.3% (*n* = 2/88) each from abscess and kidney, and 1.1% (*n* = 1/88) each from abdominal cavity, nasal swab and liver (Table 1). In order to verify the relevance of the *S. suis* isolates, the results of the histological examination were consulted. *S. suis* infection was considered as relevant in cases where the histological diagnoses included meningitis, polyarthritis, endocarditis, or placentitis, and when histological features consistent with septicemia [34] were observed. In two cases no histopathology was performed, but *S. suis* was determined relevant due to clear bacteriological findings (pure culture of *S. suis*), appropriate anamnesis and clinical signs. Isolates, for which a clear link to *S. suis* could be ruled out histopathologically (due to inappropriate age or due to another etiological diagnosis), were termed as not relevant. Isolates with an incomplete record of data, no *S. suis* relevance could be assigned and were therefore excluded for screening with the pathotyping tool.

**Table 1:** **Number of porcine*S. suis*isolates from corresponding site of isolation.**

Site of isolation	Number of isolates
Blood	30
Brain	18
Lung	13
Joint	10
Heart	7
Genital tract	3
Abscess	2
Kidney	2
Abdominal cavity	1
Nasal swab	1
Liver	1
Total	88

### DNA extraction and species identification

Isolates were cultured for 24 to 48 h at 37 °C on Columbia blood sheep agar plates (Thermo Fisher Diagnostics AG, Pratteln, Switzerland) under aerobic conditions. DNA was extracted using a standard heat lysis protocol [35]. Briefly, colony material was dissolved in lysis buffer containing Proteinase K at 60 °C for 45 min under constant shaking followed by a heat deactivation step at 96 °C for 15 min. DNA samples were diluted to 20 ng/µl for subsequent reactions.

The isolates were identified by matrix-assisted laser desorption ionization-time-of-flight mass spectrometry (MALDI-TOF MS, Bruker, Bremen, Germany). In addition, to confirm *S. suis* species, a *recN* (recombination/repair protein gene) PCR was performed as described earlier [36].

### Capsular typing and MLST

Capsular typing was performed as previously described [37]. Universal primers targeting 16S rRNA gene were used for internal control of the PCR reactions [38]. PCR products were analyzed on a capillary electrophoresis QIAxcel Advanced device (Qiagen, Hilden, Germany) using a screening cartridge, QX 15 bp–3 kb alignment marker and QX 100 bp–2.5 kb size marker (Qiagen) according to the manufacturer’s instructions. The resulting electropherograms were viewed with the QIAxcel ScreenGel 1.2.0 software (Qiagen). Isolates with capsular (*cps*) type pair 2 or 1/2 and 1 or 14 were assigned to the corresponding *cps* type using a recently developed HRM assay [39].

MLST was performed targeting *aroA, cpn60, dpr, gki, recA, thrA* and *mutS* as described previously [26]. For strains with no PCR amplification using primer mutS, alternative mutS primers have been used as described [40]. Briefly, PCR was set up in a final volume of reaction mixture of 20 µl containing HotStart*Taq* Master Mix (Qiagen), 0.5 µM of each primer pair and 20 ng template DNA with cycling conditions as described [26]. Sequencing of the seven housekeeping genes was performed by Sanger sequencing (Microsynth, Balgach, Switzerland). CLC Main Workbench 7.0.1 was used for sequence analysis. STs were assigned based on PubMLST database (https://pubmlst.org/ssuis). New housekeeping gene alleles were submitted to the database in order to create new STs. For visualizing groups of related genotypes and for identification of CCs, Swiss isolates were clustered at triple locus variant level with all isolates from the *S. suis* database (2289 isolates on September 27, 2019). The goeBURST algorithm from Phyloviz software [41] was used to visualize CCs and to create a MLST-based minimal spanning tree. CCs comprised STs with at least six identical alleles. STs that did not form any group were defined as singletons and unrelated STs grouping only with individual STs were indicated as non-defined founders.

### Virulence-associated gene profiling and MCG typing

All *S. suis* isolates were tested for three virulence-associated genes (*mrp, epf, sly*) by multiplex PCR using primers as described previously [22]. Additionally, a *epf* monoplex PCR [22] was conducted to ensure reliable detection of large variants of the *epf* gene designated as epf* [21]. To accurately differentiate size variants of *mrp*, a PCR was performed using the *mrp* variant primers as described previously [22]. The PCR assays were performed using HotStart*Taq* Master Mix (Qiagen) according to the manufacturer’s instructions. MCG sequence typing was performed as described previously [42].

### Pathotyping

Pathotyping was carried out using a recently developed multiplex PCR [31] by analyzing a selected subset of 75 isolates, which could be linked without any doubt to either etiologically relevant *S. suis* diseased or not *S. suis* relevant diseased pigs (Table 2). Briefly, a multiplex PCR was conducted using primers targeting two disease-associated genes (copper exporting ATPase 1 and type I RM system S protein), one non-disease-associated marker (putative sugar ABC transporter), and *S. suis* specific primers (sporulation regulator *WhiA*). The multiplex PCR was executed using HotStartTaq Mix (Qiagen) applying the three-step thermal cycling program as described previously [31]. PCR products were analyzed on a capillary electrophoresis QIAxcel Advanced device (Qiagen) using a screening cartridge, QX 15 bp–3 kb alignment marker and QX 100 bp–2.5 kb size marker (Qiagen) according to the manufacturer’s instructions.

**Table 2:**
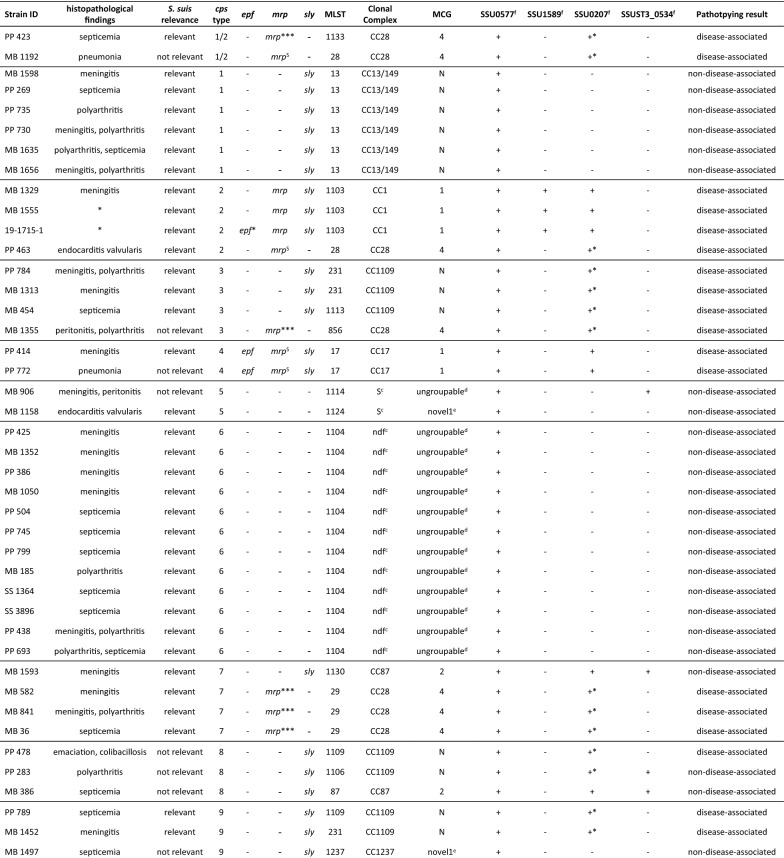

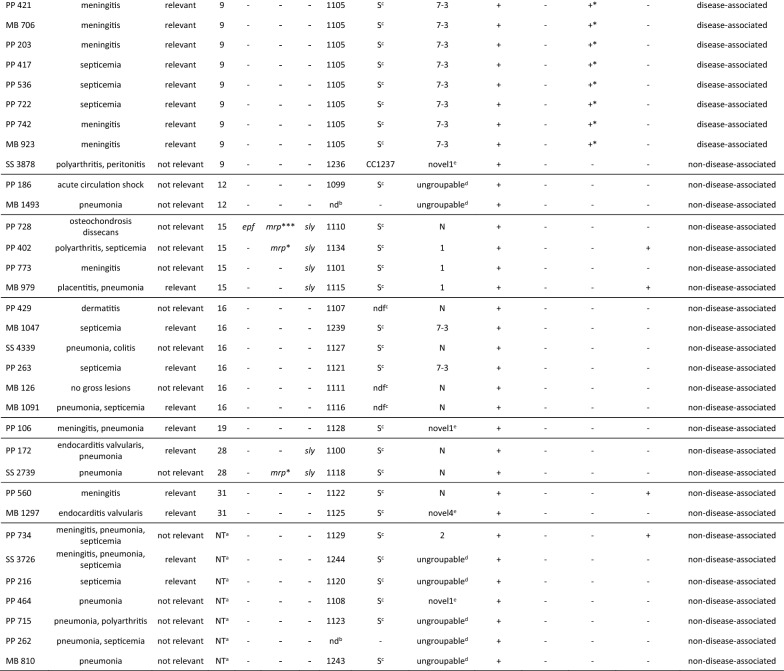
**75 porcine*S. suis*isolates evaluated with the pathotyping tool.**

Isolates were chosen based on the fact of an unequivocal link of etiologically relevant *S. suis* diseased or non-disease-associated pigs. Pathotyping results, which did not correspond to the results obtained by histopathological findings, are indicated in red.* indicates two isolates, which were included in the evaluation although a record of histopathological findings was missing, but due to evident bacteriological findings and appropriate anamnesis, strains were evaluated as relevant. *mrp* variants identified: *mrp*^S^ (747 bp), *mpr* (1148 bp), mrp* (1556 bp), mrp*** (2400 bp). epf* represents a large variant of *epf*, which was detected only in one isolate (19-1715-1). +* relates to the pathotyping tool representing a variant PCR product of copper exporting ATPase gene.

^a^Nontypeable.

^b^Not determined (nd): one lacking housekeeping gene.

^c^No clonal complex could be assigned for ST that occurred as singletons (S) or with no determined founder (ndf).

^d^Ungroupable: allele missing.

^e^Novel: allel combination not described for MCG sequencing typing by Zheng et al. [42].

^f^Wileman et al. [31].

### Whole genome sequencing of a selected panel of isolates

Whole genome sequencing (WGS) of a selected panel comprising 13 Swiss clinical *S. suis* strains of porcine origin has been performed previously [43]. Strains were chosen based on the aim to cover isolates of the most prevalent *cps* types (*cps*6 and *cps*9) and *cps* types most likely supposed to be virulent (*cps*1/2, *cps*1 and *cps*2) taking into consideration different years of sampling. Briefly, genomic DNA was isolated with the DNA Blood and Tissue Kit (Qiagen) and sequencing was performed on a MiniSeq sequencer (Illumina, San Diego, USA) with 150-bp paired-end reads using the Nextera DNA Flex kit (Illumina). Sequences are available under GenBank SRA accession numbers SRR8290472-SRR8290486.

## Results

### Pathological and bacteriological findings

In total, 88 isolates were obtained. Detailed histopathological investigations and bacteriological examinations revealed 52 isolates as *S. suis* relevant and 23 isolates as not *S. suis* relevant. For 13 isolates no detailed records were available and therefore, were not included in the comparison with the pathotyping tool. The most frequently reported history was found to be occurrence of sudden death of one or more pigs on a farm (*n* = 29/88) and observations of neurological symptoms (*n* = 24/88). In terms of post-mortem findings, the most commonly identified reason of death found by pathologists was septicemia (*n* = 27/75) and meningitis (*n* = 27/75).

### Characterization of *S. suis* isolates by *cps* typing and MLST

*Cps* typing of the isolates identified a distribution of seventeen different *cps* types comprising 89.8% of isolates, whereas in 10.2% of cases no *cps* type could be assigned and were therefore classified as nontypeable (NT) (Additional file 1). Capsular gene analysis yielded two main *cps* types: 15.9% *cps*9 (*n *= 14) and 14.8% *cps*6 (*n *= 13). Furthermore, the following *cps* types were identified: *cps*1 (*n *= 6), *cps*15 (*n *= 6), *cps*16 (*n *= 6), *cps*7 (*n *= 4), *cps*2 (*n *= 4), *cps*8 (*n *= 3), *cps*3 (*n *= 4), *cps*1/2 (*n *= 3), *cps*12 (*n *= 3), *cps*4 (*n *= 3), *cps*31 (*n *= 3), *cps*5 (*n *= 2), *cps*21 (*n *= 2), *cps*28 (*n *= 2), *cps*19 (*n *= 1), and NT (*n *= 9). Clinically observed *S. suis* relevant phenotypes confirmed by histopathological analysis was detected for isolates of *cps*1, *cps*2, *cps*6, and *cps*7. In contrast, non-relevant *S. suis* features could explicitly be associated with isolates of *cps*8.

**Figure 1:**
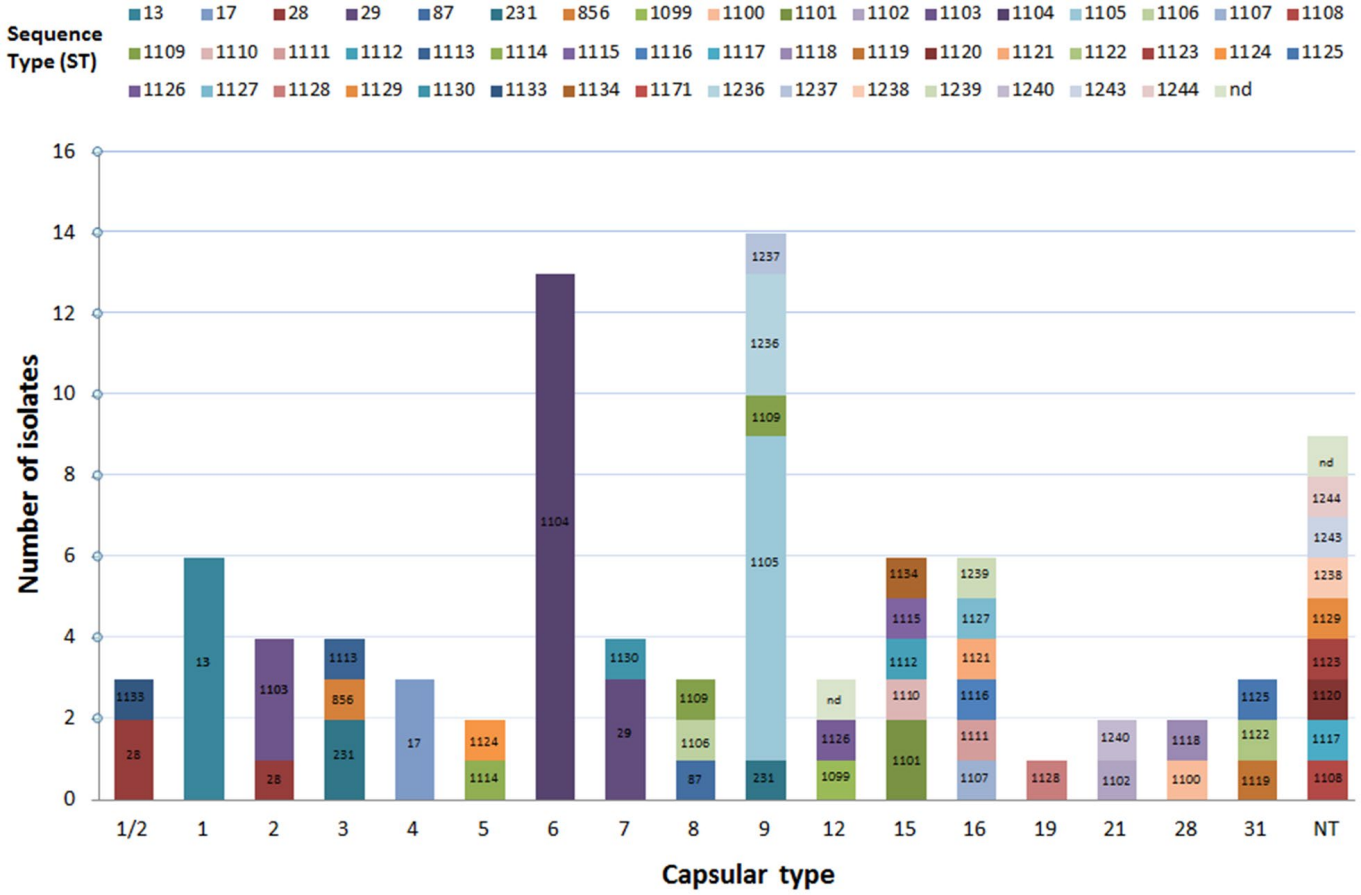
**Sequence type (ST) distribution of porcine*S. suis*in relation to capsular type.** Identified STs are shown in form of a stacked histogram and illustrated in the corresponding bar sections. Nontypeable (NT) isolates could not be identified by multiplex PCR. Isolates with one lacking housekeeping gene identification could not be determined (nd).

MLST analysis revealed that 22.7% of isolates belonged to eight previously identified STs, while 77.3% had a new MLST profile encompassing 41 new STs. The predominant STs were ST1104 (*cps*6, *n *= 13/13) comprising 14.8% of isolates, followed by 9.1% of ST1105 (*cps*9, *n *= 8/14), and 6.8% of isolates comprised ST13 (*cps*1, *n *= 6/6). The newly assigned STs in this study were ST1099–ST1130, ST1133, ST1134, ST1237–ST1240, ST1243, and ST1244 (Additional file 2). Three isolates of *cps*2 (*n *= 3/4) were an atypical single locus variant (SLV) of ST1 (ST1103), whereas one *cps*2 isolate (*n *= 1/4) was identified as a relatively common ST28 (Figure 1). The distribution of STs in relation to *cps* type highlights the diversity of *S. suis*. Thirteen of the seventeen *cps* types identified enclosed multiple STs, ranging from two to six different STs per *cps* type. *Cps*1, *cps*4, *cps*6, and *cps*19 only harbored one ST, whereas *cps*16 showed the greatest variety of six different STs (Figure 1).

### Distribution of STs in relation to year of isolation

**Figure 2:**
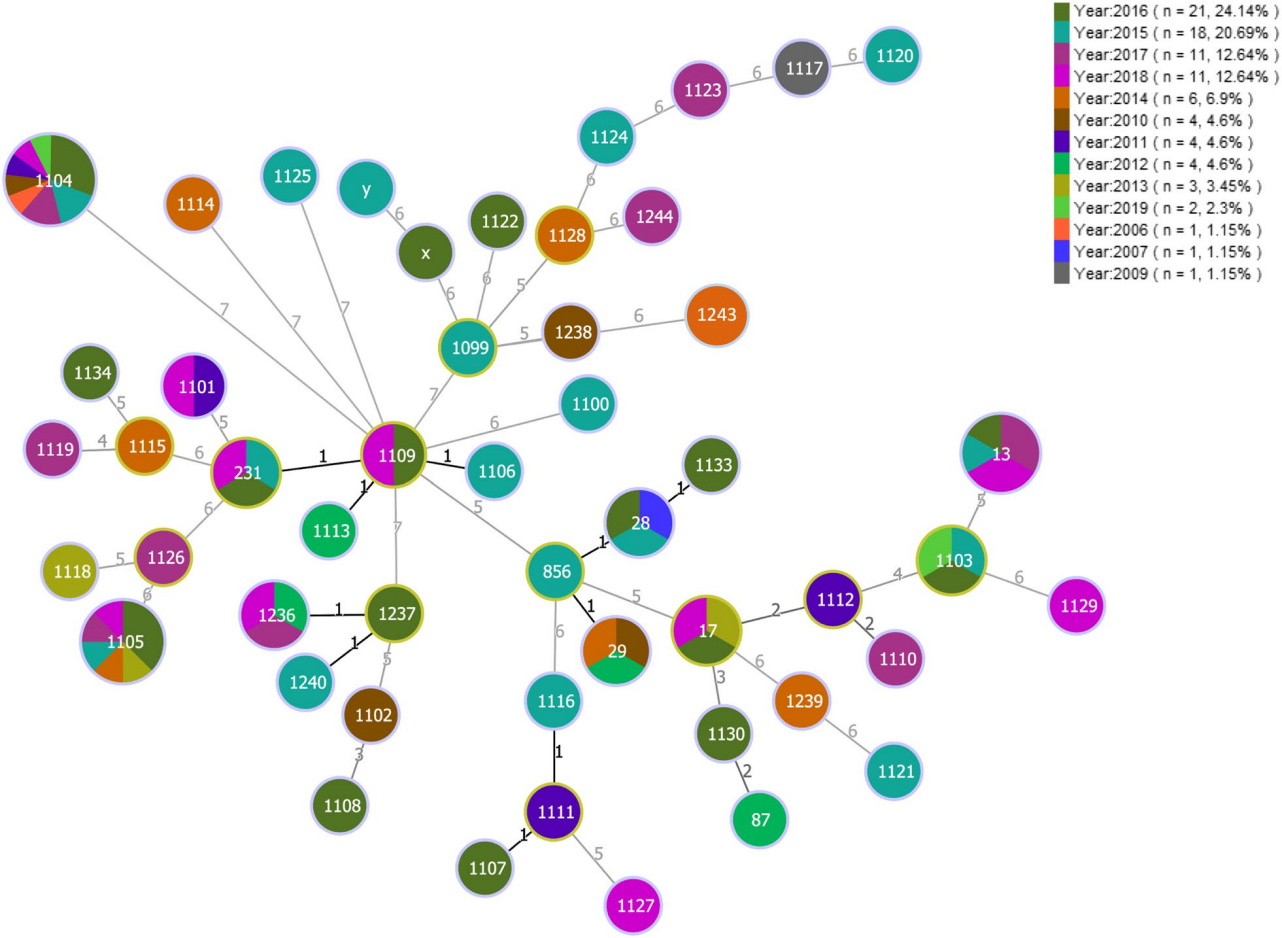
**MLST-based minimal spanning tree of 88 porcine*S. suis*isolates.** The MLST-based minimum spanning tree is representing the temporal distribution of sequence types determined for *S. suis* isolates collected during the last 13 years from diseased pigs. The tree was calculated using the goeBURST full MST algorithm in Phyloviz 2.0. Sizes of nodes reflect the number of isolates with a specific MLST profile. Numbers within the nodes indicate the corresponding sequence type. Node colors refer to the year of isolation as represented in the legend. Numbers on lines indicate locus variants between nodes. Black lines indicate single locus variants and grey lines represent multi locus variants. x and y represent isolates with no determined ST due to lacking of housekeeping gene identification.

Figure 2 illustrates a MLST-based minimal spanning tree of all tested isolates within a time span of 2006–2019. ST1104 (*cps*6, *n *= 13/13) and ST1105 (*cps*9, *n *= 8/14) were the most prevalent STs identified in respect to the temporal distribution recovered during eight and six different years, respectively. ST13 (*cps*1, *n *= 6/6) was collected in four different years. ST17 (*cps*4, *n *= 3/3), ST29 (*cps*7, *n *= 3/4), ST231 (*cps*9, *n *= 1/14; *cps*3, *n *= 2/4), ST1103 (*cps*2, *n *= 3/4), and ST1236 (*cps*9, *n *= 3/14) originated from three different years. ST1101 (*cps*15, *n *= 2/6) and ST1109 (*cps*8, *n *= 1/3; *cps*9, *n *= 1/14) were found within two different years. However, the majority of isolates, representing 43.2% of the tested collective, was isolated uniquely in 1 year.

### Association between genotype and CCs

**Figure 3:**
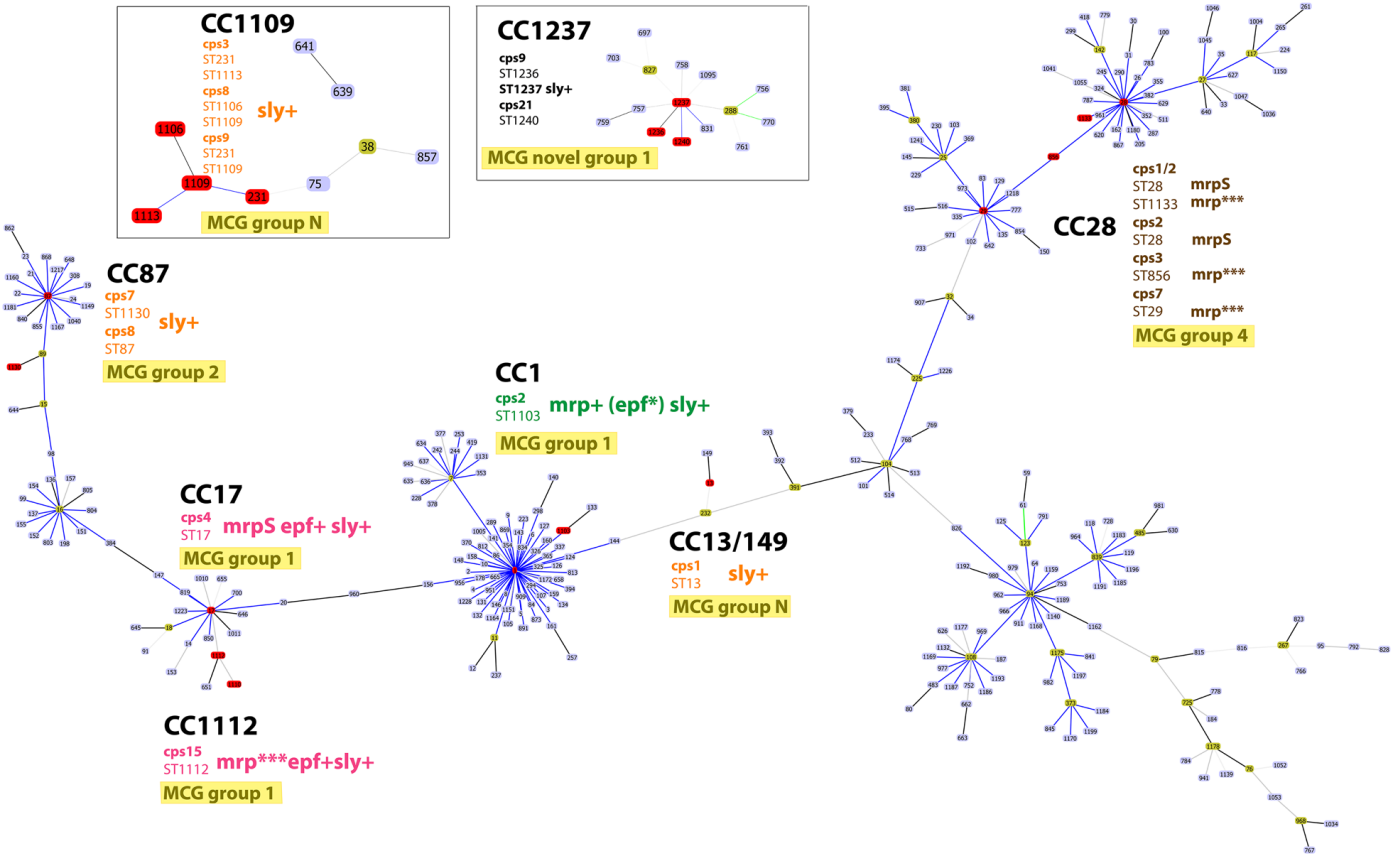
**Population snapshot of*S. suis*in Switzerland.** Groups at triple locus variants (TLV) level were created by goeBURST v1.2 software using the phyloviz software [41] applying a data set from the PubMLST database (https://pubmlst.org/ssuis). Grey lines define a link at double locus variant (DLV) or triple locus variant (TLV) between the CCs following eBURST rules. Numbers in nodes represent sequence types (ST), whereas light green represents founder groups, blue shows common nodes and red indicates STs identified in Switzerland. Clonal complexes (CCs) identified are indicated in bold. Association of Swiss isolates between capsular types (*cps*), ST and its corresponding CCs is shown. Identified virulence markers namely extracellular protein factor (*epf*), muramidase-released protein (*mrp*), and suilysin (*sly*) are indicated, highlighting its correlation with according STs and CCs. Isolates harboring *mrp *+ *epf *+ *sly *+ , *mrp *+ (*epf *+)*sly *+ , *sly *+ , and *mrp *+ are represented in pink, green, orange, and brown, respectively. Variants of *mrp* are indicated with *mrp*^S^ (small variant) and mrp*** (large variant); variant of *epf* is marked as epf* (large variant). Isolates appearing as singletons or with no determined founder are not represented. CC1109 and CC1237 are shown separately with no connections to the main CCs due to the absence of relation at TLV level. Corresponding minimum core genome (MCG) groups are highlighted in yellow.

STs of all *S. suis* isolates were assigned to CCs using the eBurst illustration (Figure 3). Among the 88 isolates analyzed, eight CCs were identified: CC1, CC17, CC28, CC87, CC13/149, CC1109, CC1112, and CC1237 comprising 3, 3, 8, 2, 6, 7, 1, and 5 isolates, respectively. The distribution of *S. suis* isolates in relation to ST revealed ST1104 (*cps*6, *n *= 13/13) with the highest prevalence of 14.8% and no association to any CCs. Further on, 9.1% of isolates representing ST1105 (*cps*9, *n *= 8/14) were found to be unrelated singleton-isolates not associated to any CCs.

A total of 6.8% of isolates belonged to CC13/149 harboring ST13 (*cps*1, *n *= 6/6). 3.4% of isolates belonged to CC28 comprising ST29 (*cps*7, *n *= 3/4). ST17, ST28, ST231, ST1103, and ST1236 represented each 3.4% of isolates associated with CC17, CC28, CC1109, CC1, and CC1237, respectively. Finally, all remaining isolates had different STs only occurring in few isolates illustrating a massive genetic diversity identified in diseased Swiss pigs (Additional file 3). Samples comprising CC1 included ST1103 (*cps*2, *n *= 3/4). ST17 (*cps*4, *n *= 3/3) was part of CC17. The most abundantly identified CC28 contained isolates of ST28 (*cps*1/2, *n *= 2/3; *cps*2, *n *= 1/4), ST29 (*cps*7, *n *= 3/4), ST856 (*cps*3, *n *= 1/4), and ST1133 (*cps*1/2, *n *= 1/3). CC87 contained ST87 (*cps*8, *n *= 1/3) and ST1130 (*cps*7, *n *= 1/4). All isolates of ST13 (*cps*1, *n *= 6/6) were part of CC13/149. CC1109 comprised isolates of ST231 (*cps*3, *n *= 2/4; *cps*9, *n *= 1/14), ST1106 (*cps*8, *n *= 1/3), ST1109 (*cps*8, *n *= 1/3; *cps*9, *n *= 1/14), and ST1113 (*cps*3, *n *= 1/4). CC1112 harbored only one isolate of ST1112 (*cps*15, *n *= 1/6). Finally, CC1237 comprised ST1236 (*cps*9, *n *= 3/14), ST1237 (*cps*9, *n *= 1/14) and ST1240 (*cps*21, *n *= 1/2) (Table 3).

**Table 3:** ***S. suis*clonal complexes (CCs) in relation to capsular type and the according sequence types (ST).**

Clonal complex (CC)	Caspular type																		Sequence Types (STs)
	1/2	1	2	3	4	5	6	7	8	9	12	15	16	19	21	28	31	NT^b^	
CC1			3																1103^d^
CC17					3														17, 1112^d^
CC28	3		1	1				3											28, 29, 856, 1133^d^
CC87								1	1										87, 1130^d^
CC13/149		6																	13
CC1109				3					2	2									231, 1106^d^, 1109^d^, 1113^d^
CC1112												1							1112^d^
CC1237										4					1				1236^d^, 1237^d^, 1240^d^
no CC^a^						2	13			8	2	5	6	1	1	2	3	8	1099-1102^d^, 1104^d^, 1105^d^, 1107^d^, 1108^d^, 1110^d^, 1111^d^, 1114-1116^d^, 1116^d^, 1117-1129^d^, 1134^d^, 1238^d^, 1239^d^, 1243^d^, 1244^d^
Ungroupable^c^											1							1	–
Total no. of isolates (n)	3	6	4	4	3	2	13	4	3	14	3	6	6	1	2	2	3	9	

^a^No CCs assigned for STs observed as singletons or with no determined founder.

^b^Nontypeable by multiplex PCR.

^c^ST could not be assigned due to failure of one housekeeping gene identification.

^d^Novel STs.

### Virulence profiling

CC1 harbored one *cps*2 isolate with a large variant of epf* (*mrp*^+^epf^*^sly^+^), whereas two *cps*2 isolates had a *mrp*^+^*epf*^−^*sly*^+^ genotype. Variable subtypes of *mrp* were identified in one *cps*2 isolate and two *cps*1/2 isolates with a genotype of *mrp*^S^*epf*^−^*sly*^−^; one *cps*1/2 isolate, one *cps*3 isolate, and three *cps*7 isolates contained mrp^***^epf^−^*sly*^−^. Furthermore, genotype *mrp*^S^*epf*^+^*sly*^+^ was found in all three isolates of *cps*4 belonging to CC17. Two isolates of *cps*15 had a mrp***epf^+^*sly*^+^ genotype, whereas one isolate of each *cps*15 and *cps*28 contained mrp^*^epf^−^*sly*^+^. 22.7% of isolates showed a *mrp*^−^*epf*^−^*sly*^+^ genotype, which was associated with CC13/149 consisting of *cps*1 (*n *= 6/6); CC1109 comprising *cps*3 (*n *= 3/4), *cps*8 (*n *= 2/3), and *cps*9 (*n *= 2/14); CC87 consisting of *cps*7 (*n *= 1/4), and *cps*8 (*n *= 1/3); CC1237 comprising *cps*9 (*n* = 1/14); and ultimately, singleton-isolates consisting of *cps*15 (*n* = 3/6) and *cps*28 (*n *= 1/2). Finally, a total of 56.8% of isolates studied had a *mrp*^−^*epf*^−^*sly*^−^ genotype, which could not be associated to any CCs and were identified as singletons consisting of *cps*5 (*n *= 2/2), *cps*6 (*n *= 13/13), *cps*9 (*n *= 11/14), *cps*12 (*n *= 3/3), *cps*16 (*n *= 6/6), *cps*19 (*n *= 1/1), *cps*21 (*n *= 2/2), *cps*31 (*n *= 3/3), and NT (*n *= 9/9) (Additional file 2).

### MCG typing

MCG typing revealed MCG group 1 for members of CC1, CC17, and CC1112; MCG group 2 for isolates of CC87; MCG group 4 for isolates of CC28; MCG group N (representing an ungroupable subset of isolates) for CC13/149 and CC1109; and a novel MCG group for isolates of CC1237. In total, 13.6% of isolates were classified into five novel MCG groups not previously described (Table 4). Almost 30% of isolates could not be assigned to any MCG group due to the failure of one housekeeping gene identification.

**Table 4:**
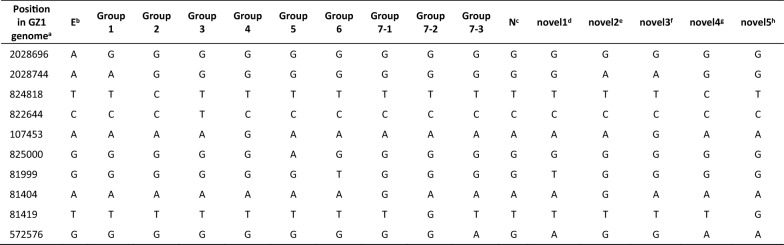
**Single nucleotide polymorphisms (SNPs) used for minimum core sequencing (MCG).**

MCG groups as defined by Zheng et al. [42] including 5 novel MCG groups identified in the present study. Red highlights discriminative SNPs for each SNP profile.

^a^Reference genome: strain GZ1 (GenBank accession number: CP000837).

^b^Epidemic (E) strains (ST7 strains) identified by Zheng et al. [42].

^c^Ungroupable.

The following isolates resulted in novel MCG groups with a new SNP profile.

^d^MB1158 (*cps* type 5), MB1497, SS2097 (*cps* type 9), PP106 (*cps* type 19), MB1185 (*cps* type 21), SS921 (nontypeable).

^e^MB1629 (*cps* type 12).

^f^Human pathogen strain (*cps* type 14).

^g^MB 1297 (*cps* type 31).

^h^SS 3919 (*cps* type 31).

### Pathotyping for differentiation between invasive disease-associated and non-disease-associated isolates

**Figure 4:**
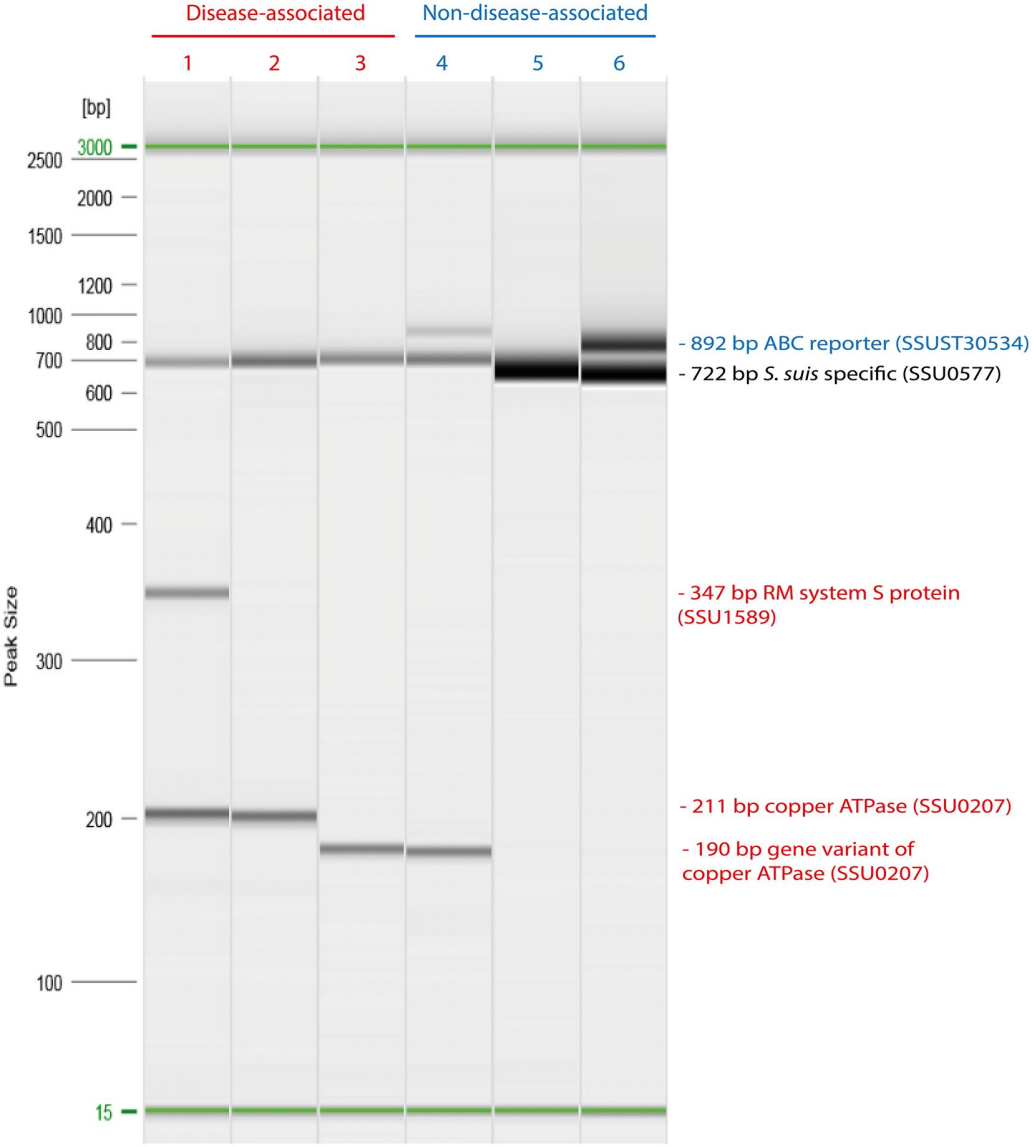
**Capillary electrophoresis plots illustrating invasive disease-associated and non-disease-associated Swiss*S. suis*isolates.** On the left hand side the DNA size marker (100 bp–2.5 kb) is shown. The alignment marker (green) is representing the start and end of electrophoresis. Amplicons sizes obtained from the molecular pathotyping tool [31] are indicated on the right hand side. Lane 1 corresponds to the amplification reaction of a highly virulent isolate of *cps* type 2 (sequence type 1103) revealing both disease-associated markers (red) predicting a putative copper exporting ATPase 1 (SSU0207) and a type I restriction-modification (RM) system S protein (SSU1589). Lane 2 and 3 represent disease-associated isolates harboring the copper ATPase and a variant form with a 21 base pair (bp) deletion (lane 3), respectively. Lane 4, 5, and 6 demonstrate observed amplification patterns of non-disease-associated samples (blue). Lane 4 shows a PCR amplicon for the putative sugar ABC reporter (SSUST30534) also in the presence of the copper ATPase gene. Samples with no amplification of any markers are considered to be non-disease-associated (lane 5). A sporulation regulator (WhiA) serves as identification control for *S. suis* (SSU0577).

Figure 4 shows the amplicon patterns obtained by multiplex PCR after capillary electrophoresis. Amplicons of *S. suis* specific genetic marker (SSU0577) were obtained by all isolates proving the correct species identification. Amplicons of 892 bp represented the putative sugar ABC reporter as a non-disease-associated marker (SSUT30534), whereas disease-associated gene markers were identified by amplicons of 347 bp and 211 bp illustrating a type I RM system S protein (SSU1589) and a predicted copper ATPase (SSU0207), respectively. Additionally, new variant forms of the copper ATPase gene marker appeared, illustrated by a 190 bp long amplicon (Additional file 4). In total, 29.3% (*n *= 22/75) of isolates analyzed harbored a gene variant of the copper ATPase gene containing partial deletions, represented by all isolates of CC28 (*n *= 7/75), CC1109 (*n *= 7/75), and *cps*9 isolates with ST1105 (*n *= 8/75). As a contrast, 4% (*n *= 3/75) of isolates belonging to CC1 showed a 211 bp long amplicon as comprehended by the virulent P1/7 strain (Table 2). The disease-associated marker type I RM system S protein-gene revealed a PCR amplification of 347 bp only for the three isolates comprising CC1. Sequence analysis of some exemplary isolates with *cps*1/2, *cps*1, and *cps*2 revealed a truncated gene version of the implicated gene, whereas the reverse primer could not bind to the target sequence due to the absence of the 3′end of the gene in most isolates analyzed (Additional file 4).

Pathotyping of the subset of 75 isolates unequivocally attributed as *S. suis* relevant or not *S. suis* relevant, revealed isolates of *cps*2 and *cps*9 to be in accordance with the pathotyping tool. However, comparing *S. suis* relevant isolates of *cps*1 and *cps*6 to results obtained by pathotyping, all isolates yielded a contradictory result. All other *cps* types could not clearly be classified by pathotyping due to the paucity of isolates having the same capsular type and therefore no statement can be made.

## Discussion

In this study a detailed assessment of genetic characteristics of Swiss *S. suis* isolates of diseased pigs was performed and compared to an earlier analyzed human isolate. In general, seventeen different *cps* types were identified underlining high serotype diversity in accordance to a previously observed massive diversity in whole genome sequences of a chosen set of isolates of clinically affected pigs [43]. The most common *cps* types found among the examined strain collection were *cps*9 and *cps*6. Contrary to a German investigation with over 700 *S. suis* isolates, not a single *cps*6 strain was found, which is worth to highlight when keeping in mind that Germany is a main neighboring country of Switzerland [44].

In the current report, all 13 isolates identified as *cps*6 comprised a single clone of ST1104, which was not associated to any CCs. ST1104 is representing a clone with no determined founder, identified to be a single locus variant (SLV; differing only at *cpn*60) of a Danish invasive *S. suis* strain with ST55 [45]. Noteworthy, this identified clone reoccurred throughout eight different years (Figure 2) underlining its capacity of causing disease in pigs due to an invasive behavior of the involved strain. Interestingly, a study from Chile, investigating phenotypically and genotypically diseased pigs at nonrelated farms during the time span of 2007–2011, revealed a single clone of *S. suis* with *n* = 28/29 of isolates identified to be serotype 6. Although an invasive phenotype could be observed, no enhanced virulence could be proven in a murine model testing serotype 6 isolates from Chile including the Danish serotype 6 reference strain. No concomitant infections of the involved isolates could be observed, therefore, an enhanced virulence capacity was suggested [46].

Furthermore, it was notable that all isolates belonging to *cps*9 were rather heterogenic harboring five different STs, although one abundant ST (ST1105, *n* = 8/14) seemed to be predominant among the identified samples. The genetic diversity of *cps*9 isolates in China and Canada showed a high variety among examined isolates [47, 48], which is comparable to the observed genetic diversity of inspected Swiss isolates. Interestingly, the aforementioned clone comprising ST1105 reoccurred within six different years (Figure 2) emphasizing its importance among the pig population in Switzerland and its ability to induce infections. Similar reports of pigs with invasive disease have stated a predominance of mrp*cps9 belonging to CC16/CC87 for Europe including The Netherlands, Spain, Germany and Belgium [9, 16]. The observation that *cps*9 isolates of the present study did not harbor *mrp* together with the identification of novel sequence types, underlines the emergence of very unique *S. suis* strains with new genetic rearrangements rarely found in other countries.

Actually, Switzerland has very little traffic of piglets with foreign countries and the pig farming system is different in comparison to other European countries. Herds are much smaller and the utilization of farrowing crates for reproduction purposes is prohibited by law since 1997. The physiological behavior in nesting is impaired due to the narrowing conditions in farrowing crates and consequently, a more natural environment helps to avoid complications during birth of piglets thereby strengthening their immune status. In addition, Switzerland is considered to be free of porcine reproductive and respiratory syndrome virus (PRRSV) [49] and *Mycoplasma hyopneumoniae* [50]. Moreover, a majority of farms vaccinate piglets against porcine circovirus (PCV) [51]. The general health status of Swiss pigs is considered to be high, which could be explained by the absence of these pathogens considered to be immunosuppressive and therefore having an implication in the defense of *S. suis*. Nevertheless, stress caused by weaning at an age of 28 days and rehousing of piglets usually after 10–12 weeks frequently paired with a change of feed at the fattening farms can lead to infection caused by virulent *S. suis* strains. Fattening farms usually combine weaning piglets of different farms leading to a higher transmission rate of *S. suis* among piglets. These circumstances could explain the high genetic variability and the distinct composition regarding ST and capsular types of *S. suis* strains obtained in this study in contrast to other countries.

Most remarkably, isolates belonging to CC1 are considered as potentially zoonotic and were mostly described as virulent strains [26]. In the present study three out of four isolates comprising *cps*2 were identified belonging to CC1. Showing its current relevance, one *cps*2 isolate originating from a brain of a diseased pig, was collected very recently in fall 2019, highlighting an emerging *S. suis* clone with ST1103. Interestingly, the implicated strain had a *mrp*^+^epf^*^sly^+^*cps*2 genotype containing a large variant of *epf*, which is considered to be a moderately virulent pathotype [21]. In fact, this specific clone is a SLV of ST1, differing only in allele *gki.* The fourth *cps*2 isolate had a more common ST28 belonging to CC28. One highly virulent human ST1 (*cps*14) isolate from another study [52] was also part of CC1 harboring *mrp*^+^*epf*^+^*sly*^+^ underlining an association between virulence-associated markers *epf*, *mrp*, and *sly* with STs rather than *cps* types (Figure 3). Three *cps*2 isolates (ST1103) were classified as MCG group 1, whereas the fourth *cps*2 isolate (ST28) was part of MCG4. For the human isolate, MCG typing revealed a novel group not described before, which was uniquely attributed to this isolate. The fact that this isolate was classified as a novel MCG group, absent among the analyzed Swiss porcine isolates, proves an improbable transmission event of this highly virulent pathogenic isolate from Swiss pigs pointing out a different source of infection. The fact that the virulent strain was introduced through a person originating from Moldova importing meat to Switzerland [52], proves an independent introduction of this zoonotic strain. Furthermore, all *cps*4 isolates which are part of CC17 were classified as MCG group 1 with a genotype of *mrp*^S^*epf*^+^*sly*^+^ emphasizing a possible increased virulence potential. In addition, a majority of *cps*15 isolates belonged to MCG group 1, whereas two isolates had a mrp^***^epf^+^*sly*^+^ genotype. Moreover, most isolates of *cps*7 were ST29 and belonged to CC28, similar to an emerging virulent strain recently reported in Germany [53] highlighting that isolates classified to be part of CC28 clearly show invasive and highly virulent features.

To evaluate the usefulness of pathotyping [31], a subset of 75 isolates having an unequivocal link of etiologically relevant *S. suis* diseased or non-disease-associated pigs was screened by the pathotyping tool. *Cps*2 and *cps*9 showed a clear correlation between pathotyping results and histopathological findings. Remarkably, comparing observed phenotypes classified to be *S. suis* relevant, obtained by a combination of histopathological examinations and bacteriological analysis, to results obtained by pathotyping, isolates of *cps*1 and *cps*6 yielded a contradictory result since no gene marker indicating disease-association yielded a positive PCR result. Due to the perceived inconsistency of phenotypically observed manifestations determined to be *S. suis* relevant in contrast to the non-*S. suis* relevant results obtained with the pathotyping tool, sequence analysis of the involved gene markers (type I RM systems S protein- and copper ATPase-gene) was performed highlighting a high genetic variability of different isolates (Additional file 4). Worth mentioning, all *cps* type 1 isolates (ST13) were associated to CC13/149. Interestingly, in two previous studies [29, 54] isolates of ST13 were associated with a pathogenic phenotype and represented an indicator of virulence underlining its importance. Yet undiscovered potential virulence factors especially of *cps*1 and *cps*6 strains remain matter of further investigation.

*Cps*3, *cps*7, *cps*15, and *cps*16 showed partially contradictory results between histopathological findings and the pathotyping tool, which points out the difficulty of consistent histopathological examinations, considering the multifaceted properties of bacterial infections.

## Conclusion

With this study an expanded knowledge about the population structure and the genetic diversity of *S. suis* could be achieved. The epidemiological situation of *S. suis* in Switzerland revealed a heterogeneous composition of strains with high genetic variability belonging to several CCs. Strikingly, *cps*9 and *cps*6 were among the predominant capsular types associated with *S. suis* diseased pigs. Genetic variant forms of implicated disease-associated genes can be explained partially with frequent genomic rearrangements in *S. suis* as shown previously [27, 55]. These genetically variable and distinct rearrangements could be facilitated by the unique pig farming of Switzerland with very little transfer of piglets from other European countries, however, with a frequent transport rate of weaning piglets to fattening farms.

In view of an affordable tool as a diagnostic application to rapidly discover highly invasive *S. suis* strains and to highlight a potentially threatening zoonotic pathogen, it would be advantageous to have a reliable pathotyping tool. The screening using our well characterized strain collection showed a good feasibility of the tool for *cps*2 and *cps*9, however, for *cps*1 and *cps*6 contradictory results were obtained. Therefore, we recommend using the pathotyping tool only in case of *cps*2- and *cps*9-identification in order to differentiate between invasive disease-associated isolates and non-disease-associated isolates.

An efficient identification of highly virulent strains could help to alert public health surveillance programs to take action in line with a one health approach. Keeping track of disease-associated isolates helps to reduce the risk of zoonotic infections and will allow promoting a high standard in animal welfare programs.

## Supplementary information

**Additional file 1. Capsular type distribution among Swiss*S. suis*isolates.** Number of isolates in relation to capsular type is indicated. NT = nontypeable due to no successful capsular typing by multiplex PCR.

**Additional file 2. Clinical data, genotyping, and determination of virulence markers of Swiss porcine*S. suis*isolates (*n* = 88).**

**Additional file 3. Distribution of porcine*S. suis*isolates in relation to sequence type and clonal complex.**

**Additional file 4. Alignment of target gene sequences and the corresponding amino acid sequences used by pathotyping.** Sequence alignment of copper ATPase 1-gene (A and B) and partial gene sequence alignment of type I RM system S protein-gene (C and D) of invasive disease-associated isolates of Swiss *S. suis* in comparison to the highly virulent reference strain P1/7 are shown visualizing different gene variants and its corresponding protein sequences. Conserved, matching nucleotide residues are illustrated as blue dots, whereas red represents differences of nucleotide sequences. (A) Copper ATPase 1-gene sequences of *S. suis* PP463 (*cps*2, ST28), SS470 (*cps*1/2, ST28), PP423 (*cps*1/2, ST1133), and PP536 (*cps*9, ST1105) are represented. Primer sequences of the pathotyping tool are indicated in green. A duplication of a 54 bp long DNA segment in isolate SS470 and deletion of a 21 bp fragment in all represented Swiss isolates could be observed, illustrating a high genetic variability. (B) Corresponding amino acid sequence alignment of Copper ATPase 1 is shown. (C) RM system S protein gene sequences of *S. suis* PP463 (*cps*2, ST28), PP423 (*cps*1/2, ST1133), and PP269 (*cps*1, ST13) are represented. The forward primer is indicated in green, whereas the reverse primer could not be shown since illustrated Swiss isolates are truncated. (D) Corresponding amino acid sequence alignment of RM systems S protein is shown.

## Data Availability

All data relevant to the study are included in the article or enclosed as additional files.

## Abbreviations

*S. suis*: *Streptococcus suis*
CPS: Capsular polysaccharides
MLST: Multilocus sequence typing
MCG: Minimum core genome
ST: Sequence type
CC: Clonal complex
EPF: Extracellular protein factor
MRP: Muramidase-released protein
SLY: Suilysin
RM: Restriction-modification
ABC: ATP-binding cassette
*recN*: Recombination/repair protein gene
*n*: Number
