# Supplementary material for: Population structure, genetic diversity and pathotypes of Streptococcus suis isolated during the last 13 years from diseased pigs in Switzerland
Source: Vet Res. 2020 Jul 8;51:85. doi: 10.1186/s13567-020-00813-w (PMC7346511; doi:10.1186/s13567-020-00813-w)
Supplement: Supplementary file 4 — Additional file 4. Alignment of target gene sequences and the corresponding amino acid sequences used by pathotyping. Sequence alignment of copper ATPase 1-gene (A and B) and partial gene sequence alignment of type I RM system S protein-gene (C and D) of invasive disease-associated isolates of Swiss S. suis in comparison to the highly virulent reference strain P1/7 are shown visualizing different gene variants and its corresponding protein sequences. Conserved, matching nucleotide residues are illustrated as blue dots, whereas red represents differences of nucleotide sequences. (A) Copper ATPase 1-gene sequences of S. suis PP463 (cps2, ST28), SS470 (cps1/2, ST28), PP423 (cps1/2, ST1133), and PP536 (cps9, ST1105) are represented. Primer sequences of the pathotyping tool are indicated in green. A duplication of a 54 bp long DNA segment in isolate SS470 and deletion of a 21 bp fragment in all represented Swiss isolates could be observed, illustrating a high genetic variability. (B) Corresponding amino acid sequence alignment of Copper ATPase 1 is shown. (C) RM system S protein gene sequences of S. suis PP463 (cps2, ST28), PP423 (cps1/2, ST1133), and PP269 (cps1, ST13) are represented. The forward primer is indicated in green, whereas the reverse primer could not be shown since illustrated Swiss isolates are truncated. (D) Corresponding amino acid sequence alignment of RM systems S protein is shown. [file 13567_2020_813_MOESM4_ESM.docx]

**Additional file 4**. Alignment of pathotyping target gene sequences and the corresponding amino acid sequences

A


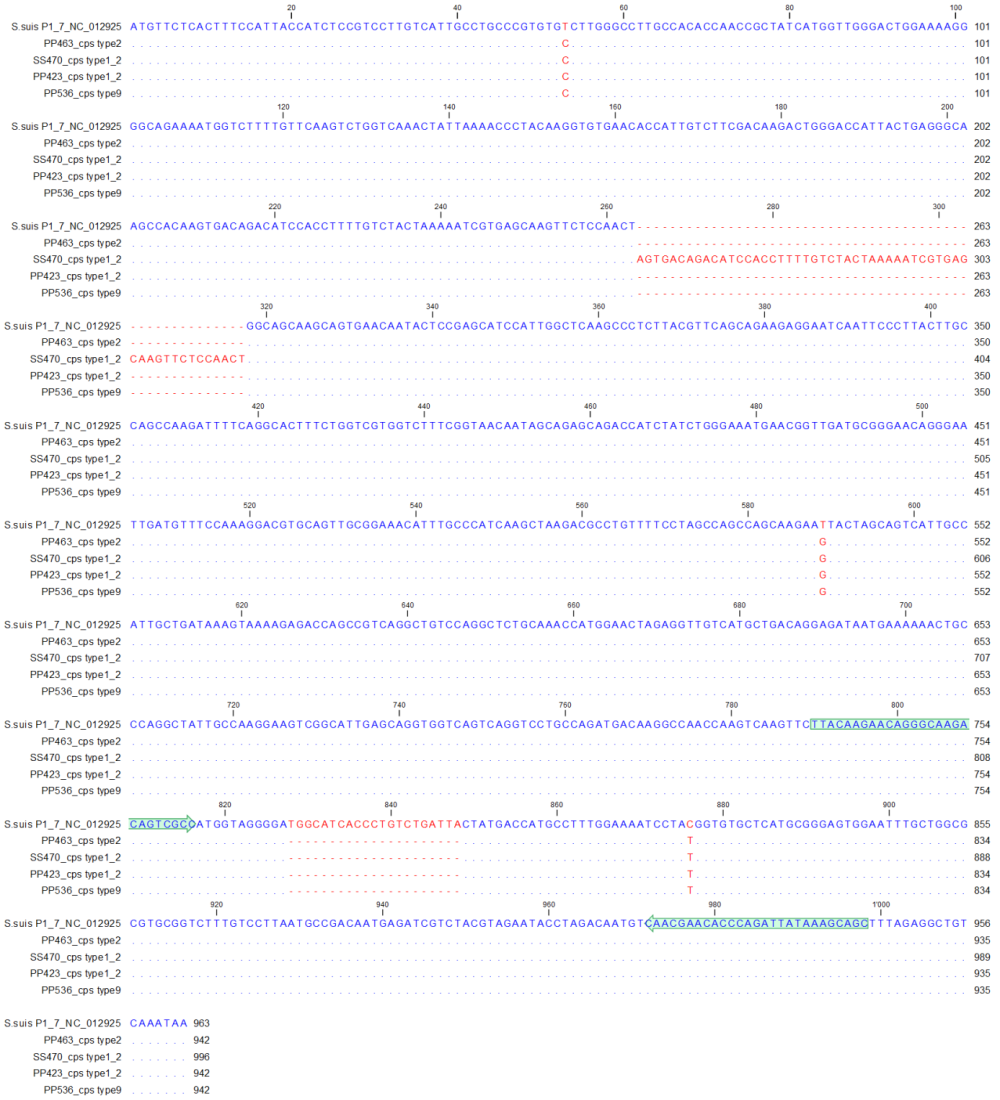


B


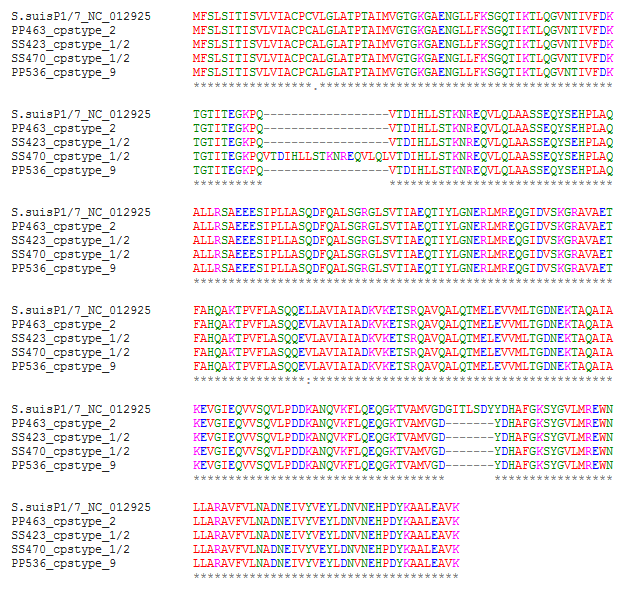


C


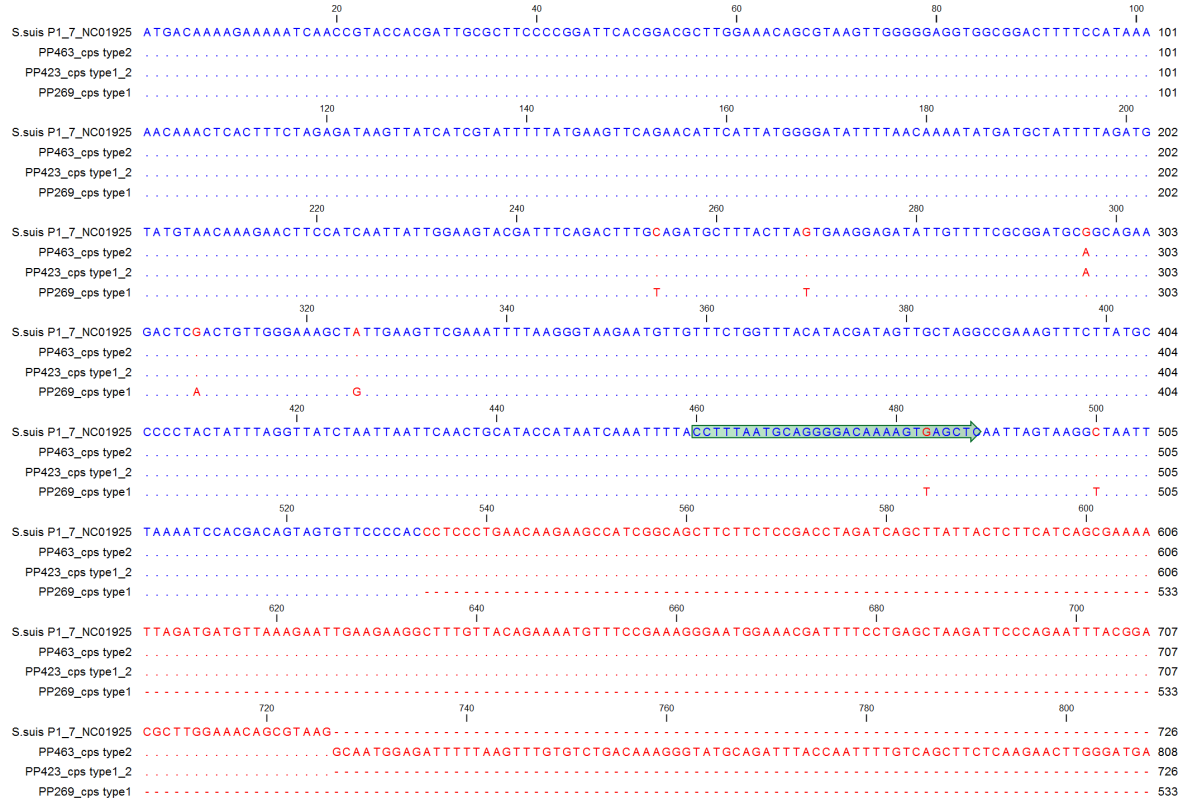


D


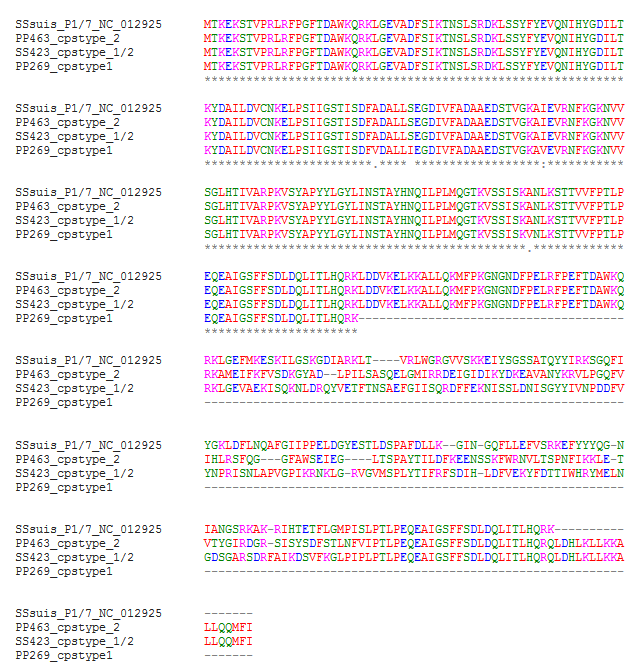


Sequence alignment of copper ATPase 1-gene (A and B) and partial gene sequence alignment of type I RM system S protein-gene (C and D) of invasive disease-associated isolates of Swiss *S. suis* in comparison to the highly virulent reference strain P1/7 are shown visualizing different gene variants and its corresponding protein sequences. Conserved, matching nucleotide residues are illustrated as blue dots, whereas red represents differences of nucleotide sequences. (A) Copper ATPase 1-gene sequences of *S. suis* PP463 (*cps* type 2, ST28), SS470 (*cps* type 1/2, ST28), PP423 (*cps* type 1/2, ST1133), and PP536 (*cps* type 9, ST1105) are represented. Primer sequences of the pathotyping tool are indicated in green. A duplication of a 54 bp long DNA segment in isolate SS470 and a deletion of a 21 bp fragment in all represented Swiss isolates could be observed, illustrating a high genetic variability. (B) Corresponding amino acid sequence alignment of Copper ATPase 1 is shown. (C) RM system S protein gene sequences of *S. suis* PP463 (*cps* 2, ST28), PP423 (*cps* 1/2, ST1133), and PP269 (*cps* 1, ST13) are represented. The forward primer is indicated in green, whereas the reverse primer could not be shown since illustrated Swiss isolates are truncated. (D) Corresponding amino acid sequence alignment of RM systems S protein is shown.
